# Der schwerwiegendste Vorfall – Erfahrungen von Aggressionen und Gewalt in der Augenheilkunde

**DOI:** 10.1007/s00347-022-01634-2

**Published:** 2022-04-20

**Authors:** C. Jacobsen, I. Volkmann, F. Wedegärtner, J. Harris, B. Bertram, B. Bambas, C. Framme

**Affiliations:** 1 Universitätsklinik für Augenheilkunde, Medizinische Hochschule Hannover (MHH), Hannover, Deutschland; 2 Universitätsklinik für Psychiatrie, Sozialpsychiatrie und Psychotherapie, MHH, Hannover, Deutschland; 3 Berufsverband der Augenärzte Deutschlands e. V., Düsseldorf, Deutschland

**Keywords:** Gewalt am Arbeitsplatz, Psychosozialer Stress, Mitarbeiterzufriedenheit, Arbeitssicherheit, Bedrohung, Workplace violence, Psychosocial stress, Employee satisfaction, Work safety, Threats

## Abstract

**Hintergrund:**

Erfahrungen von Aggressionen/Gewalt beeinflussen die Arbeitszufriedenheit und können die Mitarbeiter psychisch und physisch langfristig belasten. Im Herbst 2018 führten der Berufsverband der Augenärzte Deutschlands e. V. (BVA) und die Deutsche Ophthalmologische Gesellschaft e. V. (DOG) eine Umfrage zu Erfahrungen von Aggressionen und Gewalt durch. Die ersten Ergebnisse wurden 2020 veröffentlicht. In der Umfrage konnte zudem der bislang schwerwiegendste Vorfall unter anderem durch Freitextfelder geschildert werden.

**Methodik:**

Alle 9411 Mitglieder von DOG und BVA erhielten 2018 die Möglichkeit, online einen Fragebogen zu Aggressionen und Gewalt in der Augenheilkunde auszufüllen.

**Ergebnisse:**

Es berichteten 253 von 1508 (16,8 %) an der Umfrage teilgenommenen Ophthalmologen über ihren schwerwiegendsten Vorfall, der zu 46,8 % als mittelschwer eingestuft wurde und zu 34,3 % verbale Gewalt wie Beleidigung und Bedrohung umfasste. Den schwerwiegendsten Vorfall erlebten 171 (67,6 %) Ärzte in einer Praxis; 71 % waren zum Tatzeitpunkt Fachärzte. Die Vorfälle ereigneten sich zu 74,3 % in der regulären Arbeitszeit. Ursachen waren v. a. interkulturelle Konflikte, lange Wartezeiten, Probleme bei der Terminvergabe, zu hohe Erwartungshaltung, Behandlungsdifferenzen oder eine Grundaggressivität. Dabei waren die Täter zu 86,3 % männlich. Bei 15,8 % der Vorfälle erfolgte eine polizeiliche Meldung; 21 (8,3 %) Ärzte erteilten einen Praxisverweis oder Hausverbot.

**Diskussion:**

Die Schilderung der schwerwiegendsten Vorfälle veranschaulicht mitunter kaum vorstellbare Situationen und auch, welche Vorfälle als gravierend eingeschätzt wurden. Es existieren dabei große subjektive Schwankungen in der Beurteilung der Vorfälle. Schutzmaßnahmen in Praxen und Kliniken sind unerlässlich.

## Abstract (en)

**Background:**

Experiences of aggression/violence influence job satisfaction and can have a long-term psychological and physical impact on employees. In the fall of 2018, the Professional Association of Ophthalmologists (BVA) and the German Ophthalmological Society (DOG) conducted a survey on experiences of aggression and violence. The first results were published in 2020. In the survey it was also possible to describe the most serious incident to date using free text fields, among others.

**Method:**

All 9411 members of the DOG and BVA were given the opportunity to complete a questionnaire online in 2018 regarding aggression and violence in ophthalmology.

**Results:**

Overall, 253 of 1508 (16.8%) ophthalmologists participating in the survey reported their most serious incident, 46.8% of which were classified as moderate and 34.3% were related to verbal violence such as insults and threats. The most serious incident was experienced by 171 (67.6%) physicians in a practice setting, 71% were specialists at the time of the incident and 74.3% of the incidents occurred during regular working hours. The main causes were intercultural conflicts, long waiting times, problems with the allocation of appointments, excessive expectations, differences in treatment and basic aggressiveness. The offenders were male in 86.3% of cases, 15.8% of the incidents were reported to the police and 21 (8.3%) physicians issued a practice reprimand or house ban.

**Discussion:**

The description of the most serious incidents illustrates situations that are sometimes hard to imagine and also which incidents were considered serious. There are large subjective variations in the assessment of the incidents. Protective measures in practices and clinics are essential.

## Hintergrund

Erfahrungen von Aggressionen und Gewalt im Beruf beeinflussen nicht nur die Arbeitszufriedenheit, sondern üben darüber hinaus einen großen Einfluss auf die Psyche der Betroffenen aus. So verwundert es nicht, dass Gewalterfahrungen im Beruf mit einem höheren Risiko verbunden sind, ein Burn-out zu erleiden [1]. Aus diesen Gründen erscheint es notwendig, sowohl die Aggressionen und Gewalterfahrungen in der Medizin systematisch zu erfassen, als auch die Faktoren zu analysieren, die für diese Situationen ursächlich sind. Auf der Basis dieser Erkenntnisse können Maßnahmen zur Anpassung und Optimierung der Rahmenbedingungen entwickelt und vorgenommen werden, die zu einer Minderung von Episoden mit Aggressionen und Gewalterfahrungen führen.

Im Herbst 2018 führten der Berufsverband der Augenärzte Deutschlands e. V. (BVA) und die Deutsche Ophthalmologische Gesellschaft e. V. (DOG) eine Umfrage unter ihren Mitgliedern zu ihren Erfahrungen mit Aggressionen und Gewalt durch. Die ersten Ergebnisse dieser Umfrage wurden 2020 veröffentlicht. Dabei bejahten 83,3 % der 1508 Augenärztinnen und Augenärzte, die sich an der Umfrage beteiligten, bereits Aggressionen/Gewalt im Rahmen ihrer ärztlichen Tätigkeit erfahren zu haben. Insbesondere Augenärztinnen sowie junge Ärztinnen und Ärzte meldeten signifikant häufiger entsprechende Erfahrungen [2]; 65,6 % der Umfrageteilnehmer berichteten von verbalen Übergriffen ohne Drohung wie Beleidigungen, Fluchen, Beschimpfungen oder Schreien. Der Mittelwert der Anzahl der in den vergangenen 12 Monaten erlebten Vorfälle lag hier bei 10,7 Vorfällen. Über körperliche Gewalterfahrungen berichteten 24,1 % der Teilnehmenden. Sexuelle Übergriffe mit dem Charakter einer Einschüchterung oder Belästigung widerfuhren 322 (21,4 %) der Befragten.

Im Rahmen der Umfrage konnten die Teilnehmer zudem Angaben zu dem jeweils schwerwiegendsten Vorfall angeben, den sie bislang erlebt hatten, und in Freitextfeldern nähere Angaben machen. Mit diesem Manuskript werden diese zusätzlichen subjektiven Angaben quantitativ und qualitativ ausgewertet. Die Ergebnisse liefern einen Überblick über die jeweiligen Vorfälle und zeigen, wie Aggressionserfahrungen im beruflichen Kontext von den Opfern bewertet werden. Mit den ermittelten Angaben zu den Hintergründen der Vorfälle können ferner Maßnahmen zur Prävention entworfen und eingeleitet werden.

## Methodik

Über die Webplattform Soscisurvey (SoSciSurvey, SoSci Survey GmbH, München, www.soscisurvey.de, Server: sosci01.mh-hannover.local, Version 3.2.00) konnten im Herbst 2018 alle 9411 Mitglieder von DOG und BVA online einen Fragebogen zur Aggressionen und Gewalt ausfüllen. Grundlage für den Fragebogen waren die Aggressions-Wahrnehmungsskala (POPAS-Fragebogen) [3] sowie die Umfrage zu Aggressionen und Gewalt unter Allgemeinmedizinern [4]. Personalisierte Einladungen mit Token stellten sicher, dass Teilnehmer nur jeweils 1‑mal teilnehmen konnten. Sämtliche Daten wurden nach Abschluss der Befragung vollständig anonymisiert.

Die statistische Auswertung und die Generierung von Tabellen, Grafiken und Datenlisten wurden mit dem statistischen Programm SPSS Version 25.0 (Armonk, NY, USA: IBM Corp) sowie Excel (Version 2019, Microsoft, Redmond, WA, USA) durchgeführt. Es wurden Subgruppen für verschiedene Erfahrungen unter anderem nach Geschlecht und subjektiver Einschätzung der Schwere des Vorfalls gebildet. Die Freitextfelder zur Schilderung des Vorfalls sowie möglicher Ursachen wurden einzeln analysiert und nach eigener Einschätzung in vergleichbare Aussagen gruppiert.

## Ergebnisse

**Figure 1:**
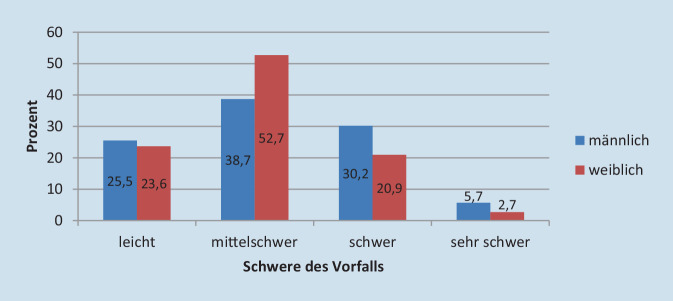

Es berichteten 253 von 1508 (16,8 %) Augenärztinnen und Augenärzten über die als schwerwiegendsten Vorfall wahrgenommene persönliche Erfahrung, wobei dieser subjektiv als „leicht“ über „mittelschwer“ und „schwer“ bis hin zu „sehr schwer“ kategorisiert werden konnte. Der Anteil von Frauen an diesem Kollektiv betrug 58,9 %. Ärztinnen und Ärzte erfuhren zu 46,8 % mittelschwere Erlebnisse (Abb. 1).

Den schlimmsten Vorfall erlebten 172 (68,8 %) Ärzte in einer Praxis, davon 84 (33,6 %) in Einzelpraxen, 67 in Gemeinschaftspraxen (26,8 %) und 21 in medizinischen Versorgungszentren (8,4 %). In 44 (17,6 %) Fällen ereignete sich der jeweils schwerwiegendste Vorfall in einer nicht-universitären Klinik und 34 (13,6 %) Fälle in einer Universitätsklinik.

Zum Tatzeitpunkt bezeichneten sich 176 (71,0 %) als Fachärzte (5,2 % Chefärzte, 1,2 % leitende Oberärzte, 4,4 % Oberärzte und 60,1 % sonstige Fachärzte) und 72 (29,0 %) Ärzte befanden sich in der Weiterbildung. 188 (74,3 %) Vorfälle ereigneten sich in der regulären Arbeitszeit mit 150 (59,3 %) in Praxen und 38 (15,0 %) in Kliniken; 48 (19 %) Ärzte erlebten nachts die schlimmsten Erfahrungen, wovon 34 (13,4 %) in Kliniken passierten. Nur 17 (6,7 %) der Vorfälle geschahen am Wochenende oder an Feiertagen.

### Art des Vorfalls

**Figure 2:**
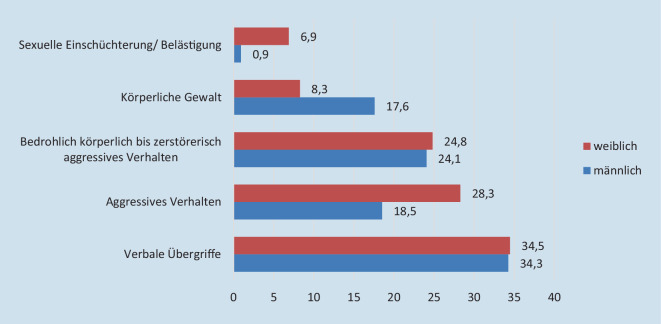

Verbale Gewalt wie Beleidigung und Bedrohung wurde in 34,3 % als schwerwiegendster Vorfall beurteilt (Abb. 2). Körperliche Gewalt widerfuhr v. a. den männlichen Augenärzten. Dagegen nannten Ärztinnen vermehrt sexuelle Einschüchterung/Gewalt sowie aggressives Verhalten als schlimmstes Ereignis.

Die leichten Vorfälle entfielen zu 38,7 % auf die Kategorie verbaler Übergriffe ohne Drohung und waren hier besonders Fluchen und Schreien. Ursachen waren unter anderem Behandlungsdifferenzen und eine zu hohe Erwartungshaltung. So wurde z. B. eine notwendige Änderung der Glaukomtherapie nicht verstanden und vehement abgelehnt. Öfter führten als unangemessen empfundene Wartezeiten zu den Vorfällen. In einem anderen Fall kam es zu einer sexuellen Anspielung, falls eine anstehende Operation nicht erfolgreich verlaufen würde.

Die mittelschweren Vorfälle waren zu 20,2 % bedrohendes verbales Verhalten. In einem Fall wurde beispielsweise mit Erschießung gedroht, sollte kein zeitnaher Behandlungstermin eingeräumt werden. Andere Patienten wurden aggressiv, als keine betäubenden Augentropfen oder Opiate gegeben wurden.

Schwere Vorfälle wurden zu 28,6 % als bedrohlich körperliches Verhalten eingestuft. So zertrümmerte ein gesunder Patient eine Glasschale am Tresen, da ihm die Ausstellung einer Arbeitsunfähigkeitsbescheinigung verweigert wurde, die der Patient gefordert hatte, weil seine Brille zerbrochen war und die Wartezeit auf Ersatz eine Woche betrug. Öfter wurde Gewalt angedroht, falls eine Behandlung nicht den erwarteten erfolgreichen Verlauf nehmen würde. Ein Angehöriger drohte am Telefon, dass er bei Misslingen der Operation seines Vaters mit der „Knarre vorbeikommen“ würde. Hier zeigt sich bereits, dass die Einschätzung des Schweregrads der Vorfälle durch die Umfrageteilnehmer sehr subjektiv erfolgte.

Insgesamt wurden 10 (4,0 %) Vorfälle als sehr schwer eingestuft. Bei 3 (1,2 %) dieser Vorfälle handelte es sich um bedrohendes verbales Verhalten, im Rahmen dessen 2 Ärzten mit dem Tod gedroht wurde, weil Unterschiede bei der Einschätzung der Behandlungsnotwendigkeit vorlagen. Ein Arzt berichtete von einem bedrohlich körperlichen Verhalten eines Patienten, in dessen Verlauf dieser ihm einen Revolver an die Schläfe hielt, weil die Schmerzen einer Erosio corneae aus Sicht des Patienten nicht schnell genug gelindert wurden. Ein anderer Arzt wurde an einem Samstagabend mit einem Messer bedroht, um der Forderung nach sofortiger Behandlung Nachdruck zu verleihen. Bei 3 (1,2 %) der als schwerwiegendsten Vorfälle geschilderten Vorgänge kam es zu schwerer körperlicher Gewalt. Hierbei kam es in einem Fall zu einer Geiselnahme in der Oberarztkabine und in einem anderen Fall zu einem geplanten Raubüberfall mit einem Toten.

Vereinzelt wurde von Vorfällen berichtet, in denen Ärzte v. a. unter Aggressionen durch Kollegen litten. So klagte ein Arzt über aggressives Verhalten von Vorgesetzten in Form von als unangemessen empfundenem schreiendem Zurechtweisen sowie der Herabwürdigung in Anwesenheit von Assistenzpersonal. Ein anderer Augenarzt berichtete, dass er unter psychischer Gewalt leide, die von in der Hierarchie über ihm stehenden ärztlichen Kollegen ausginge.

Berichtet wurde auch über Patienten, die mit schlechten Internetbewertungen, Medien oder Anwälten gedroht haben, falls die Forderungen der Patienten/Angehörigen nicht erfüllt wurden. Ein anderer Augenarzt fühlte sich durch anonyme Diffamierungen im Internet sehr belastet, gegen die man sich nicht wehren könne, und fürchtete reale Begegnungen mit den Verfassern solcher Bewertungen.

### Ursachen

In vielen schwerwiegenden Fällen waren die Konflikte interkultureller Natur mit rassistischen Beleidigungen seitens der Patienten/Angehörigen oder dem Vorwurf des rassistischen Verhaltens durch das Personal.

Mehrfach kam es zu Auseinandersetzungen, weil Patienten sich aus kulturellen bzw. religiösen Gründen von Ärztinnen nicht behandeln lassen wollten und auf die Behandlung durch männliche Ärzte bestanden. In einem Fall drohte ein Angehöriger, er würde eine behandelnde Ärztin „abstechen“, falls die Untersuchung nicht von einem männlichen Kollegen fortgesetzt würde, denn eine (weibliche) Ärztin sei nicht kompetent und kein „richtiger“ Arzt.

Es wurde 32-mal (12,6 %) in den Freitextfeldern die Wartezeit als Ursache für den Vorfall genannt. In 13 (5,1 %) Fällen führte die Terminvergabe zu Konflikten. In 13 (5,1 %) Fällen wurde eine Grundaggressivität des Täters als ursächlich beschrieben.

Ein Umfrageteilnehmer beschrieb das hohe Konfliktpotenzial durch die Diskussion um individuelle Gesundheitsleistungen.

### Der typische Täter

**Figure 3:**
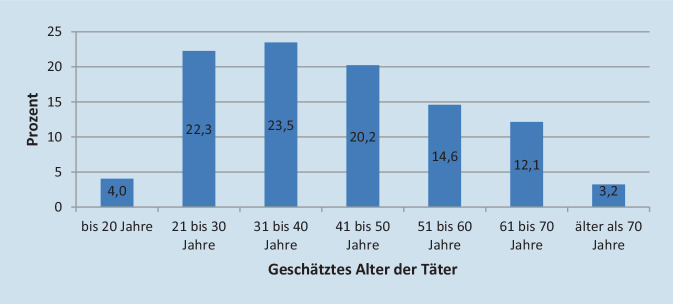

Die Täter waren zu 87,3 % männlich und zu 66,0 % zwischen 20 und 50 Jahre alt (Abb. 3). Die Täter waren zu 70,5 % Patienten und zu 21,1 % Angehörige; 32,4 % der Täter sollen laut Einschätzung der Augenärzte einen Migrationshintergrund gehabt haben. Bewusstseinsverändernde Faktoren wie Alkohol, Drogen oder psychische Faktoren lagen bei 17,8 % der Täter vor. Dabei war Alkoholkonsum bei 6,7 %, psychische Faktoren waren bei 6,7 % und Drogen bei 4,3 % der schlimmsten Fälle laut Einschätzung der Augenärzte involviert. In 32 % der Fälle konnten die Ärzte keine Aussagen diesbezüglich treffen.

### Folgen des Vorfalls

Bei 90 (35,6 %) Ärzten hatte der Vorfall keine Folgen. Nach dem Vorfall sprachen jedoch 54 (21,3 %) Ärzte mit Angehörigen, Kollegen oder Freunden über das Erlebte. Drei (1,2 %) Ärzte wurden körperlich verletzt. Eine polizeiliche Meldung erfolgte bei 40 (15,8 %) Vorfällen. Allerdings gab es auch 2 Fälle (0,8 %), in denen von der Polizei von einer Anzeige abgeraten wurde, um weitere Konsequenzen wie Rachehandlungen zu verhindern, da die Täter entsprechend polizeilich bekannt waren. In ärztliche oder psychologische Behandlung mussten 8 (3,2 %) Umfrageteilnehmer; 21 (8,3 %) Ärzte erteilten einen Praxisverweis oder Hausverbot. In 8 Fällen (3,2 %) wurden Schutzmaßnahmen eingeführt. So werden Notdienste nicht mehr allein durchgeführt, Überwachungskameras angebracht oder sogar Wachhunde eingesetzt.

## Diskussion

Viele Augenärzte haben in ihrer Tätigkeit bereits Aggressionen/Gewalt erfahren. Dabei existiert v. a. im Bereich der verbalen Gewalt ein großer Ermessensspielraum. Die Auswertung der Freitextfelder verdeutlicht, welche Situationen die Augenärzte erlebten und was die befragten Ärzte als schwerwiegend einstuften. Hier zeigten sich große subjektive Schwankungen in der Einschätzung des Schweregrads der Vorfälle. Ähnliches wurde von Dresen et al. wahrgenommen, die Umfragen mit Freitextfeldern unter Pädiatern in den Jahren 2009 und 2017 analysierten. Beispielsweise wurde das „am Kittel festhalten“ nicht nur als angedrohte Gewalt, sondern auch als versuchte Gewaltanwendung oder als ausgeübte Gewalt bewertet [5].

In der Umfrage von Vorderwülbecke unter Allgemeinmedizinern äußerten sich 449 von 831 Teilnehmern zum gravierendsten Vorfall. Dabei fanden 68 % der Vorfälle in der regulären Arbeitszeit statt im Vergleich zu 74,3 % in dieser Umfrage; 121 Schilderungen betrafen Beleidigungen und Bedrohungen, und 119 Vorfälle beinhalteten Gewalt. In 90 (20 %) Fällen kam es zu einer Meldung bzw. Anzeige bei der Polizei ähnlich wie unter den Ophthalmologen. In beiden Umfragen wurden 4 % der Vorfälle als sehr schwer eingestuft [4].

In einer Umfrage zu Gewalt in Notaufnahmen 2018 im Rahmen des Forschungsprojekts GINA wurden als Ursache von Aggression und/oder Gewalt Alkohol- oder Drogeneinfluss (86 %), lange Wartezeiten (83 %), Verwirrtheit der Patienten (54 %), Unzufriedenheit mit der Versorgung (45 %) und Verständigungsprobleme (38 %) genannt [6]. Gerade in den Freitextfeldern wurde in dieser Umfrage öfter auf lange Wartezeiten oder Unzufriedenheit mit der Versorgung hingewiesen. Mit zunehmendem Versorgungsmangel ist hier eine Zuspitzung der Situation zu erwarten [7].

In dieser Analyse wurden v. a. Extremfälle als Ergänzung zu der primär quantitativen Analyse in der ersten Auswertung erwähnt, die bereits 2020 erschien [2]. Die Limitationen von qualitativen Umfragen müssen beachtet werden. Auch ob die genannten Vorfälle tatsächlich so stattgefunden haben, kann nicht überprüft werden.

In der Zwischenzeit hat v. a. die COVID-19-Pandemie den Alltag in den Kliniken und Praxen maßgeblich verändert. Viele Einrichtungen versuchen seitdem aus Hygienegründen die Anzahl der Personen vor Ort zu reduzieren durch beispielsweise das Verbot von Begleitpersonen oder optimierte Praxisabläufe zur Reduktion von Wartezeiten. Gerade die Wartezeit führte in unserer Befragung öfter zu Auseinandersetzungen. Auf der anderen Seite führte die COVID-19-Pandemie zu anderen Konfliktsituationen, wenn z. B. das Tragen eines Mund-Nasen-Schutzes verweigert wird oder Besuchsverbote existieren. Auch hat es die Terminvergabe oft nicht vereinfacht. Die Problematik der zunehmenden Aggression insbesondere durch COVID-19 thematisierte auch das *Deutsche Ärzteblatt* im Dezember 2021. Demnach nehmen sich immer mehr Ärztekammern dem Thema an [8, 9]. In der Befragung unter Pädiatern kam es v. a. zu Beschimpfungen mit dem Vorwurf der fachlichen Inkompetenz sowie Beleidigungen. Dies kann zu persönlicher Belastung führen mit Angst vor Diensten, Schlaflosigkeit, Ärger, Aufregung, Wut sowie psychischen und psychosomatischen Beschwerden [4].

Hüpfner beschrieb 2020 Konzepte gegen Gewalt im Krankenhaus [10]. Hierbei ist insbesondere auf die Grundsatzerklärung zum Schutz vor Gewalt in der Notaufnahme, modifiziert nach dem American College of Emergency Physicians, zu verweisen [11]. Vorbeugende Schutzmaßnahmen können baulich-technische (wie Rückzugsräume, klare Fluchtwege, Sprechzimmer mit 2 Türen, keine toten Winkel oder Videoüberwachung), organisatorische (wie Gefährdungsbeurteilungen und Abstimmungen über Verhalten bei Aggressionsfällen) oder personenbezogene (Schulungen) Maßnahmen sein [12]. Da die Augenheilkunde v. a. ambulant Patienten versorgt, zeigen unsere Daten, wie gefährlich die ambulante Versorgung sein kann. Praxen sind im Wesentlichen offen für jedermann. Für kleinere Einrichtungen sind viele Maßnahmen relativ aufwendig, doch sind gerade dort viele Mitarbeiter allein tätig und einem größeren Risiko ausgesetzt, sodass das Thema nicht vernachlässigt werden sollte. Hier sollte jedoch auch die Zulässigkeit der Maßnahmen sichergestellt sein [13]. Dabei können auch kleine Handlungen zur Gewaltprävention beitragen, indem z. B. das Abnehmen der Jacke angeboten wird, da sich Waffen eher dort befinden können. Bislang gibt es sehr wenige Untersuchungen zu Aggressionen/Gewalt in der Augenheilkunde. Im J2021 wurde eine Umfrage unter Augenärztinnen und Augenärzten in Australien und Neuseeland zu Mobbing, Belästigung und sexueller Diskriminierung veröffentlicht [14]. Diese basierte auf Umfragen aus 2015 und 2018, in denen 37,6 % (2015) und 49,2 % (2018) der Umfrageteilnehmer entsprechende Erfahrungen gemacht haben. Während in unserer Umfrage primär Aggressionen/Gewalt seitens Patienten/Angehörigen erfragt wurden, handelte es sich hierbei um Missstände im Arbeitsumfeld. Allerdings wurde auch in Deutschland vereinzelt über Aggressionen/Gewalt durch Kollegen berichtet. Da bieten sich weitere Analysen für die Situation in Deutschland an.

Aufgrund der Tatsache, dass – vielleicht auch vermehrt unter den Belastungen der COVID-19-Pandemie – sich nicht nur der Pflegemangel weiter verstärkt, sondern auch der Ärztemangel dazu beiträgt, dass es offensichtlich immer schwieriger wird, Termine beim Facharzt zu bekommen und/oder adäquate Wartezeiten bei der Konsultation zu erreichen, ergibt sich weiteres Potenzial für Aggressionen gegen Ärzte. Die Daten zeigen die teilweise erhebliche Schwere der Übergriffe, sodass man die Thematik eines adäquaten Mitarbeiterschutzes in Klinik und Praxis immer ernster nehmen muss.

## Fazit

- Viele Augenärzte erlebten Gewalt/Aggressionen in ihrer Arbeit. Es existieren jedoch große subjektive Schwankungen in der Einschätzung der Schwere von Vorfällen und der daraus folgenden persönlichen Belastung für die Mitarbeiter.
- Multifaktorielle Ursachen führten zu den Vorfällen. Der zunehmende Fachkräftemangel sowie die COVID-19-Pandemie können die Situation verschlechtern.
- Gerade ambulante Einrichtungen sind oft ungefiltert Gewalt/Aggressionen ausgesetzt und haben wenig Schutzmaßnahmen.
- Augenärztliche Einrichtungen sollten Maßnahmen zur Gewaltprävention ergreifen.

## Anhang

**Table 1:**

*Bitte erinnern Sie sich an den schwerwiegendsten aggressiven Vorfall während Ihrer Tätigkeit als Arzt/Ärztin in der Augenheilkunde. Sollten Sie bislang keine Gewalt in der Augenheilkunde erfahren haben, gehen Sie bitte zum nächsten Teil der Umfrage*	
**1. Wie schwerwiegend war der Vorfall Ihrer Meinung nach?**	☐ Leicht ☐ Mittelschwer ☐ Schwer ☐ Sehr schwer
**2. Aus welcher Kategorie der in Teil A genannten Erfahrungen war der Vorfall?**	☐ Verbale Übergriffe ohne Drohung ☐ Bedrohendes verbales Verhalten ☐ Demütigendes aggressives Verhalten ☐ Herausforderndes aggressives Verhalten ☐ Passiv aggressives Verhalten ☐ Spaltend aggressives Verhalten ☐ Bedrohlich körperliches Verhalten ☐ Zerstörerisch aggressives Verhalten ☐ Mäßige körperliche Gewalt ☐ Schwere körperliche Gewalt ☐ Mäßige gegen sich selbst gerichtete Gewalt ☐ Schwere gegen sich selbst gerichtete Gewalt ☐ Versuchter Suizid ☐ Vollendeter Suizid ☐ Sexuelle Einschüchterung/ Belästigung ☐ Sexueller Übergriff
**3. Wo hat sich der Vorfall ereignet?**	☐ Praxis ☐ Klinik
**4. Wann hat sich der Vorfall ereignet?**	☐ Reguläre Arbeitszeit ☐ Nachts ☐ Wochenende/Feiertag
**5. Trat der Vorfall im Notdienst auf (siehe auch Teil B)?**	☐ Ja ☐ Nein
**6. Welches Geschlecht hatte der Täter/die Täterin?**	☐ Männlich ☐ Weiblich
**7. Welches Alter hatte der Täter/die Täterin ungefähr?**	________ in Jahren
**8. War der Täter/die Täterin Patient/-in oder Angehörige(r)?**	☐ Patient/-in ☐ Angehörige(r) ☐ Sonstiges: _______
**9. Hatte der Täter/die Täterin einen Migrationshintergrund?**	☐ Ja ☐ Nein ☐ Unbekannt
**10. Lagen bewusstseinsverändernde Faktoren beim Täter/bei der Täterin vor?**	☐ Nein ☐ Unbekannt ☐ Alkohol ☐ Drogen ☐ Psychische Faktoren
**11. Können Sie sich vorstellen, weshalb es zu dem Vorfall kommen konnte?**	Freitext
**12. Hatte dieser Vorfall Folgen?**	☐ Nein ☐ Gespräch mit Angehörigen, Kollegen, Freunden ☐ Körperliche Verletzung ☐ Meldung bei der Polizei/Anzeige ☐ Ärztliche Behandlung ☐ Psychologische Behandlung ☐ Sonstiges: ____________
**13. Möchten Sie den Vorfall genauer schildern?**	☐ Nein ☐ Ja → Bitte nutzen Sie das Freitextfeld
